# Boosting with AIDSVAX B/E Enhances Env Constant Region 1 and 2 Antibody-Dependent Cellular Cytotoxicity Breadth and Potency

**DOI:** 10.1128/JVI.01120-19

**Published:** 2020-01-31

**Authors:** David Easterhoff, Justin Pollara, Kan Luo, William D. Tolbert, Brianna Young, Dieter Mielke, Shalini Jha, Robert J. O’Connell, Sandhya Vasan, Jerome Kim, Nelson L. Michael, Jean-Louis Excler, Merlin L. Robb, Supachai Rerks-Ngarm, Jaranit Kaewkungwal, Punnee Pitisuttithum, Sorachai Nitayaphan, Faruk Sinangil, James Tartaglia, Sanjay Phogat, Thomas B. Kepler, S. Munir Alam, Kevin Wiehe, Kevin O. Saunders, David C. Montefiori, Georgia D. Tomaras, M. Anthony Moody, Marzena Pazgier, Barton F. Haynes, Guido Ferrari

**Affiliations:** a Duke University, Durham, North Carolina, USA; b U.S. Military HIV Research Program, Walter Reed Army Institute of Research, Silver Spring, Maryland, USA; c U.S. Army Medical Directorate, AFRIMS, Bangkok, Thailand; d Boston University, Boston, Massachusetts, USA; e Thai Ministry of Public Health, Nonthaburi, Thailand; f Mahidol University, Bangkok, Thailand; g Royal Thai Army Component, AFRIMS, Bangkok, Thailand; h Global Solutions of Infectious Diseases, South San Francisco, California, USA; i Sanofi Pasteur, Swiftwater, Pennsylvania, USA; j The Henry M. Jackson Foundation for the Advancement of Military Medicine, Bethesda, Maryland, USA; k Department of Biochemistry and Molecular Biology, University of Maryland, Baltimore, Maryland, USA; l Infectious Diseases Division, Uniformed Services University of the Health Sciences, Bethesda, Maryland, USA; Icahn School of Medicine at Mount Sinai

**Keywords:** HIV vaccine, antibody function

## Abstract

Induction of protective antibodies is a critical goal of HIV-1 vaccine development. One strategy is to induce nonneutralizing antibodies (NNAbs) that kill virus-infected cells, as these antibody specificities have been implicated in slowing HIV-1 disease progression and in protection. HIV-1 Env constant region 1 and 2 (C1C2) monoclonal antibodies (MAbs) frequently mediate potent antibody-dependent cellular cytotoxicity (ADCC), making them an important vaccine target. Here, we explore the effect of delayed and repetitive boosting of RV144 vaccine recipients with AIDSVAX B/E on the C1C2-specific MAb repertoire. It was found that boosting increased clonal lineage-specific ADCC breadth and potency. A ligand crystal structure of a vaccine-induced broad and potent ADCC-mediating C1C2-specific MAb showed that it bound a highly conserved Env gp120 epitope. Thus, boosting to affinity mature these types of IgG C1C2-specific antibody responses may be one method by which to make an improved HIV vaccine with higher efficacy than that seen in the RV144 trial.

**IMPORTANCE** Over one million people become infected with HIV-1 each year, making the development of an efficacious HIV-1 vaccine an important unmet medical need. The RV144 human HIV-1 vaccine regimen is the only HIV-1 clinical trial to date to demonstrate vaccine efficacy. An area of focus has been on identifying ways by which to improve upon RV144 vaccine efficacy. The RV305 HIV-1 vaccine regimen was a follow-up boost of RV144 vaccine recipients that occurred 6 to 8 years after the conclusion of RV144. Our study focused on the effect of delayed boosting in humans on the vaccine-induced Env constant region 1 and 2 (C1C2)-specific antibody repertoire. It was found that boosting with an HIV-1 Env vaccine increased C1C2-specific antibody-dependent cellular cytotoxicity potency and breadth.

## Abstract (precis)

Over one million people become infected with HIV-1 each year, making the development of an efficacious HIV-1 vaccine an important unmet medical need. The RV144 human HIV-1 vaccine regimen is the only HIV-1 clinical trial to date to demonstrate vaccine efficacy. An area of focus has been on identifying ways by which to improve upon RV144 vaccine efficacy. The RV305 HIV-1 vaccine regimen was a follow-up boost of RV144 vaccine recipients that occurred 6 to 8 years after the conclusion of RV144. Our study focused on the effect of delayed boosting in humans on the vaccine-induced Env constant region 1 and 2 (C1C2)-specific antibody repertoire. It was found that boosting with an HIV-1 Env vaccine increased C1C2-specific antibody-dependent cellular cytotoxicity potency and breadth.

## INTRODUCTION

CD4-inducible (CD4i) epitopes within HIV-1 envelope (Env) constant regions 1 and 2 (C1C2) are targets for antibodies that mediate antibody-dependent cellular cytotoxicity (ADCC) (1). C1C2-specific antibody epitopes have been termed cluster A (1) and defined by two Env-targeted monoclonal antibodies (MAbs), A32 (2) and C11 (1). Structural analyses of antigen complexes formed by A32, A32-like (3–5), and C11-like (6) MAbs indicate that these MAbs bind distinct Env epitopes. The A32 epitope involves a discontinuous sequence within Env layers 1 and 2 of the inner domain (4, 5), while the C11 epitope maps to the inner domain eight-stranded β sandwich (6). Importantly, both MAbs are nonneutralizing for tier 2 HIV strains but are capable of broad and potent ADCC (1, 2).

The secondary analysis of HIV-1 infection risk in RV144 (ClinicalTrials registration no. NCT00223080) indicated that ADCC in the presence of low anti-Env IgA responses correlated with decreased HIV-1 acquisition (7). While antibodies representative of the Env variable region 2 (V2) response inversely correlated with HIV-1 acquisition (7), we previously demonstrated that synergy between A32-blockable C1C2-specific MAbs and V2-specific MAbs increased ADCC potency of the V2 MAbs induced in the RV144 trial (8).

Here, we studied the effect of late boosting of RV144 vaccinees in the RV305 HIV-1 vaccine trial (ClinicalTrials registration no. NCT01435135), focusing specifically on C1C2-specific MAb affinity maturation, ADCC potency, and ADCC breadth. We found that the RV144 ALVAC/AIDSVAX B/E immunization regimen induced durable C1C2-specific memory B cells and that boosting with AIDSVAX B/E could increase C1C2-specific MAb variable heavy and variable light (V_H_ + V_L_) chain gene mutation frequency along with increasing ADCC breadth and potency.

(This article was submitted to an online preprint archive [9]).

## RESULTS

### AIDSVAX B/E N-terminal deletion alters C1C2-specific antibody responses.

**FIG 1:**
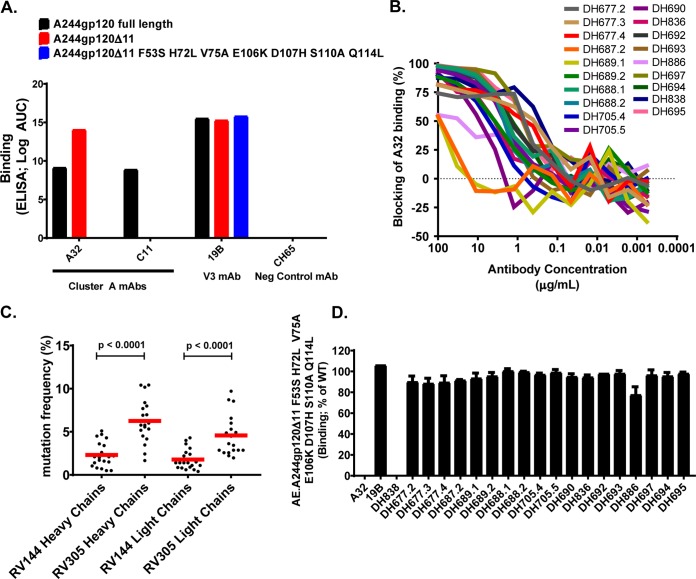
Identification of RV305 C1C2-specific MAbs. (A) The C1C2-specific MAbs A32 and C11 were assayed by ELISA for reactivity with full-length AE.A244gp120 or AE.A244g120Δ11. An A32-specific mutant protein was designed (AE.A244g120Δ11 F53S H72L V75A E106K D107H S110A Q114L) to identify A32-like MAb responses. 19B was used as a positive control and CH65 as a negative control. (B) RV305 nonneutralizing MAbs were assayed for A32 blocking by ELISA. (C) RV305 nonneutralizing A32-blockable MAb heavy- and light-chain gene sequence mutation frequencies were analyzed by Cloanalyst (13) and compared to previously published RV144 heavy- and light-chain gene sequence mutation frequencies (percent nucleotide) (12). Statistical significance was determined using a Wilcoxon rank sum test. Red bar represents the means. (D) RV305 nonneutralizing A32-blockable MAbs were assayed by ELISA for binding to AE.A244g120Δ11 and AE.A244g120Δ11 F53S H72L V75A E106K D107H S110A Q114L. Data are expressed as percent binding of the mutant protein relative to the wild type. Shown are the means with standard deviations from two independent experiments.

The AIDSVAX B/E protein used in the RV144 and RV305 HIV-1 vaccine trials had an eleven-amino-acid (aa) N-terminal deletion (10) that removed a majority of the C11-like MAb epitope (6), whereas the CRF_01 AE gp140 Env 92TH023 in ALVAC (vCP1521) retained the gp120 N-terminal 11 amino acids (11). To determine if C11 could bind to gp120 proteins with an 11-aa N-terminal deletion, we assayed A32 and C11 MAbs for binding to full-length AE.A244gp120 or to AE.A244gp120Δ11 (N-terminal 11 aa deleted). A32 bound to full-length AE.A244gp120, and binding was enhanced on AE.A244gp120Δ11 (Fig. 1A) (10). In contrast, C11 only bound to the full-length AE.A244gp120 (Fig. 1A). From these data we concluded that C11-like antibody responses were unlikely to be boosted by AIDSVAX B/E.

Peripheral blood mononuclear cells (PBMCs) collected from four vaccine recipients 2 weeks after the second RV305 boost with AIDSVAX B/E (RV305 group II) were used for AE.A244gp120-specific single B cell sorting and antibody-variable region reverse transcription-PCR (RT-PCR). A total of 19 RV305-derived nonneutralizing antibodies (NNAbs) were identified that blocked the C1C2 MAb A32 binding to AE.A244gp120Δ11 (Fig. 1B and Table 1). Compared to previously published RV144 C1C2-specific MAbs (12), the RV305 C1C2-specific MAbs had significantly more V_H_ and V_L_ chain gene mutations (*P* < 0.0001 by Wilcoxon rank sum test) (Fig. 1C), suggesting that RV305 boosting induced additional somatic mutations in C1C2-specific antibodies.

**TABLE 1:** Immunogenetics of nonneutralizing A32-blocking RV305 C1C2-specific MAbs^*a*^

Study	Visit	PTID	DH	Parameters for:								
				Heavy chains					Light chains			
				Variable segment	Joining segment	CDR3 length (aa)	% mut (nt)	Ig isotype/subclass	Variable segment	Joining segment	CDR3 length (aa)	% mut (nt)
RV144	8	RV144_140	DH677.1	1∼8	1	16	1.04	IgG1	κ1∼27	5	9	0.857
RV305	5	RV305_094	DH677.3	1∼8	1	16	3.47	IgG1	κ1∼27	5	9	2.87
RV305	5	RV305_082	DH697	1∼46	6	24	7.99	IgG1	λ1∼44	3	11	4.87
RV305	5	RV305_094	DH677.2	1∼8	1	16	2.43	IgG1	κ1∼27	5	9	2.86
RV305	5	RV305_082	DH838	3∼23	4	12	4.17	IgG1	Λ8∼61	3	10	2.22
RV305	5	RV305_094	DH677.4	1∼8	1	16	4.51	IgG1	κ1∼27	5	9	2.57
RV305	5	RV305_082	DH695	1∼2	6	22	5.56	IgG1	κ1∼39	3	9	5.30
RV305	5	RV305_082	DH694	1∼46	6	22	10.42	IgG1	κ1∼39	5	9	8.71
RV305	5	RV305_094	DH688.1	1∼46	6	23	6.97	IgG3	κ1∼39	4	9	6.00
RV305	5	RV305_094	DH705.5	1∼46	6	23	5.77	IgG3	κ3∼20	2	10	4.49
RV305	5	RV305_094	DH705.4	1∼46	6	23	6.01	IgG1	κ1∼39	5	9	3.43
RV305	5	RV305_3119	DH886	1∼2	4	12	8.38	IgG3	κ1∼39	4	9	4.92
RV305	5	RV305_031	DH690	1∼46	6	24	6.92	IgG1	λ1∼44	2	11	3.62
RV305	5	RV305_094	DH692	1∼46	6	25	1.66	IgG1	κ1∼39	4	9	2.57
RV305	5	RV305_094	DH693	1∼46	4	26	5.18	IgG1	κ1∼9	5	9	2.86
RV305	5	RV305_094	DH836	1∼46	6	16	5.32	IgG1	κ1∼12	4	9	2.86
RV305	5	RV305_094	DH688.2	1∼46	6	23	7.69	IgG3	κ1∼39	4	9	6.57
RV305	5	RV305_031	DH689.1	1∼46	6	22	10.17	IgG3	κ1∼39	2	9	9.71
RV305	5	RV305_031	DH689.2	1∼46	6	22	10.41	IgG3	κ1∼39	1	8	8.55
RV305	5	RV305_031	DH687.2	1∼46	4	23	5.77	IgG1	κ3∼20	3	10	1.97

a PBMCs used for MAb isolation were collected 2 weeks after the final boost in RV144 or 2 weeks after the second boost in RV305. All four RV305 vaccinees studied were from group II, boosted 2× with AIDSVAX B/E. RT-PCR-amplified variable heavy- and light-chain genes were Sanger sequenced (Genewiz) and analyzed with Cloanalyst (13). PTID, patient identifier; DH, antibody identifier.

To determine if the RV305-boosted A32 blockable MAbs contained a binding epitope similar to that of A32, we used the A32 ligand crystal structure (5) to identify critical A32 antibody contact residues and then designed an AE.A244gp120Δ11 mutant protein (AE.A244gp120Δ11 F53S, H72L, V75A, E106K, D107H, S110A, Q114L) to eliminate A32 MAb binding (Fig. 1A). In an enzyme-linked immunosorbent assay (ELISA), the RV305 antibody, DH838, was the only MAb with binding eliminated by mutating the A32 epitope (Fig. 1D). Likewise, DH838 was the only MAb that used a VH3 family gene, while all other ALVAC/AIDSVAX B/E-induced C1C2-specific MAbs used VH1 genes (Table 1). Thus, as in RV144, AIDSVAX B/E boosting preferentially expanded C1C2-specific antibodies that used VH1 family genes (12). A majority of the MAbs assayed in this study bound epitopes distinct from A32 but in close enough proximity to be sterically cross-blocked by A32 (Fig. 1B).

### Boosting increased C1C2-specific ADCC breadth and potency.

RV305 C1C2-specific MAbs and a subset of RV144 C1C2-specific MAbs were next assessed for ADCC against CEM.NKR_CCR5_ cells infected with one of seven HIV-1 infectious molecular clones (IMCs), representing three different clades (HIV-1 AE.CM235, B.WITO, C.TV-1. C.MW965, C.1086C, C.DU151, and C.DU422) (Table 2). These IMCs were chosen because they represent clusters of IMCs with different sensitivity to ADCC (unpublished data). To ascertain ADCC breadth and potency, MAbs were ranked using an ADCC score (see Materials and Methods) analogous to calculating MAb neutralization breadth. Apart from the RV144-derived A32 blockable MAb CH38, which was naturally an IgA MAb but tested here as a recombinant IgG1 MAb, ADCC scores for 16/19 RV305 MAbs ranked higher than those of the RV144 MAbs (Tables 3 and 4).

**TABLE 2:** Properties and subtypes of viruses used in luciferase and infected cell elimination assays

Virus name	Subtype	IMC type	Assay used
1086c	C	Luciferase Env-IMC	Luciferase
CM235	AE	Luciferase Env-IMC	Luciferase
Du151	C	Luciferase Env-IMC	Luciferase
Du422	C	Luciferase Env-IMC	Luciferase
MW96.5	C	Luciferase Env-IMC	Luciferase
TV-1	C	Luciferase Env-IMC	Luciferase
WITO	B	Luciferase Env-IMC	Luciferase
CH77	B	Full-length IMC	Infected cell elimination assay
CH264	C	Full-length IMC	Infected cell elimination assay
CH0470	B	Full-length IMC	Infected cell elimination assay
CH042	C	Full-length IMC	Infected cell elimination assay
CH185	C	Full-length IMC	Infected cell elimination assay
CH162	C	Full-length IMC	Infected cell elimination assay
CH236	C	Full-length IMC	Infected cell elimination assay

**TABLE 3:** C1C2-specific ADCC of infectious molecular clone-infected cells

Antibody	Study	IMC-infected cell ADCC endpoint concn (μg/ml)						
		AE.CM235	B.WITO	C.TV-1	C.MW96.5	C1086.C	C.DU151	C.DU422
A32		0.0006104	0.002097	0.0006104	0.009766	0.0009997	0.001628	0.009766
CH38	RV144	0.0006104	0.00675	0.001343	0.01723	0.008663	0.0333	>40
CH57	RV144	0.008608	>40	5.368	>40	9.307	0.1427	7.142
CH90	RV144	0.06479	23	1.281	>40	>40	0.4033	4.474
CH54	RV144	0.08913	1.079	0.1192	>40	1.944	0.1169	>40
DH677.1	RV144	0.008816	1.01	>40	>40	>40	>40	>40
DH677.3	RV305	0.0007527	0.006146	0.002376	6.151	0.01439	0.02068	0.02528
DH697	RV305	0.004967	0.03909	0.02134	0.0657	0.08982	0.1096	1.047
DH677.2	RV305	0.002912	0.02705	0.01351	>40	0.03581	0.5741	>40
DH838	RV305	0.0006104	0.008903	0.03242	>40	0.005042	>40	>40
DH677.4	RV305	0.00182	0.008885	0.03447	6.947	0.02838	0.3027	>40
DH695	RV305	0.005664	0.5867	0.07879	1.325	0.3076	0.3342	12.15
DH694	RV305	0.0091	0.1159	0.1249	>40	0.2651	1.854	>40
DH688.1	RV305	0.03942	0.09033	0.1375	8.079	0.3554	1.268	>40
DH705.5	RV305	0.0122	0.07738	0.09824	>40	0.08681	0.3366	>40
DH705.4	RV305	0.03119	0.2424	0.1735	2.813	0.1999	1.8	>40
DH886	RV305	0.01321	0.1425	0.1367	>40	1.583	0.1721	>40
DH690	RV305	0.09713	0.1758	0.3679	7.525	0.6221	2.862	4.34
DH692	RV305	0.07932	0.1438	0.3851	5.228	0.1444	6.399	>40
DH693	RV305	0.01995	2.168	0.05216	0.8583	1.361	2.194	6.363
DH836	RV305	0.02868	0.6244	0.1854	>40	0.1397	12.02	>40
DH688.2	RV305	0.009277	0.1436	0.1338	>40	0.5658	4.409	>40
DH691	RV305	0.03596	0.9201	0.465	8.113	0.6149	2.376	>40
DH689.1	RV305	0.352	1.629	1.69	>40	4.817	9.348	30.49
DH689.2	RV305	0.3429	2.225	0.4746	>40	5.814	6.567	1.094
DH687.2	RV305	>40	>40	20.61	>40	2.033	>40	>40

**TABLE 4:** Ranking C1C2-specific MAbs by ADCC breadth and potency^*a*^

Rank	Antibody	Study	Score	No. of strains recognized
1	A32		6.62	7
2	CH38	RV144	6.28	6
3	DH677.3	RV305	4.56	7
4	DH697	RV305	2.70	7
5	DH677.2	RV305	1.72	5
6	DH838	RV305	1.62	4
7	DH677.4	RV305	1.30	6
8	DH695	RV305	0.86	7
9	DH688.1	RV305	0.66	6
10	DH694	RV305	0.58	5
11	DH705.5	RV305	0.52	5
12	DH705.4	RV305	0.46	6
13	DH690	RV305	0.36	7
14	DH692	RV305	0.08	6
15	DH693	RV305	0.06	7
16	DH886	RV305	−0.08	5
17	DH688.2	RV305	−0.30	5
18	DH836	RV305	−0.32	5
19	CH57	RV144	−0.48	5
20	CH90	RV144	−1.06	5
21	CH54	RV144	−1.20	5
22	DH689.2	RV305	−1.42	6
23	DH689.1	RV305	−1.68	6
24	DH677.1	RV144	−2.20	2
25	DH687.2	RV305	−2.70	2

a RV305 and RV144 C1C2-specific MAbs were assayed for antibody-dependent cellular cytotoxicity against AE.CM235, B.WITO, C.TV-1, C.MW965, C.1086C, C.DU151, and C.DU422 infectious molecular clone-infected CEM.NKR_CCR5_ cells. MAbs were ranked using an ADCC score that accounts for breadth and potency (see Materials and Methods). Number of strains recognized was determined by ADCC endpoint concentration.

### Boosting of RV144 vaccine recipients with AIDSVAX B/E in the RV305 trial increased ADCC breadth and potency of the RV144-derived C1C2-specific, DH677 clonal lineage.

**FIG 2:**
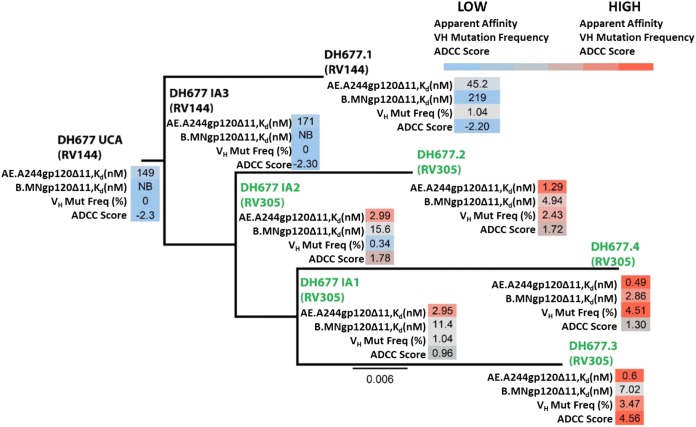
RV305 boosting increased the apparent affinity and antibody-dependent cellular cytotoxicity breadth and potency of the C1C2-specific RV144-derived DH677 memory B cell clonal lineage. DH677.1 was isolated by AE.A244gp120Δ11-specific single-cell sorting of PBMCs collected from a vaccinee 2 weeks after the final boost in the RV144 vaccine trial. DH677.2, DH677.3, and DH677.4 were isolated by AE.A244gp120Δ11-specific single-cell sorting of PBMCs collected from the same vaccinee after the second AIDSVAX B/E (RV305 group II) boost given in RV305 (∼7 years later). The intermediate ancestor one (IA1), intermediate ancestor two (IA2), intermediate ancestor three (IA3), and unmutated common ancestor (UCA) were inferred using Cloanalyst (13). The MAbs were recombinantly expressed and assayed by surface plasmon resonance for binding to the AIDSVAX B/E proteins AE.A244g120 full length, AE.A244g120Δ11, and B.MNg120Δ11. Shown are the antibody apparent affinity measurements (*K_d_*), expressed in nanomolars. NB, no detectable binding. MAbs were also assayed for ADCC against AE.C235-, B.WITO-, C.TV-1-, C.MW965-, C.1086C-, C.DU151-, and C.DU422-infected CEM.NKR_CCR5_ cells. An ADCC score (see Materials and Methods) was used to account for ADCC breadth and potency.

The C1C2-specific DH677 memory B cell clonal lineage next was used to study affinity maturation and ontogeny of AIDSVAX B/E-induced ADCC responses. B cell clonal lineage member DH677.1 was isolated from PBMCs collected from a vaccine recipient 2 weeks after the last boost in the original RV144 trial (ALVAC plus AIDSVAX B/E). DH677.2, DH677.3, and DH677.4 clonal lineage members were isolated from PBMCs collected from the same vaccine recipient 2 weeks after the second boost with AIDSVAX B/E alone given in the RV305 clinical trial (RV305 group II). Thus, this B cell clonal lineage belongs to a long-lived memory B cell pool started by the RV144 vaccine regimen and boosted many years later with the RV305 vaccine regimen (Fig. 2). DH677.1, DH677.2, DH677.3, and DH677.4 MAb sequences were used to infer with Cloanalyst (13) three intermediate ancestors (IA), IA1, IA2, and IA3, and an unmutated common ancestor (UCA) for the DH677 clonal lineage. The DH677 clonal lineage then was assayed by surface plasmon resonance for binding to the AIDSVAX B/E proteins, AE.A244gp120Δ11 and B.MNgp120Δ11, as well as full-length AE.A244gp120. The DH677 UCA did not bind to B.MNgp120Δ11 and had minimal binding to the full-length AE.A244gp120, and this binding was enhanced with AE.A244gp120Δ11 (Fig. 2). The RV305 boosts more than doubled the V_H_ chain gene mutation frequency from 1.04% (DH677.1; RV144) up to 4.51% (DH677.4; RV305), which resulted in a 100-fold increase in apparent affinity (*K_d_*) for the AIDSVAX B/E proteins (DH677.1 AE.A244gp120Δ11, *K_d_* = 45.2 nM; B.MNgp120Δ11, *K_d_* = 219 nM; DH677.4 AE.A244gp120Δ11, *K_d_* = 0.49 nM; B.MNgp120Δ11, *K_d_* = 2.86 nM) and also improved binding to full-length AE.A244gp120 (DH677.1 AE.A244gp120 full length, *K_d_* = 152 nM; DH677.4, *K_d_* = 2.29 nM) (Fig. 2).

The ontogeny of vaccine-induced ADCC was studied by assaying the DH677 clonal lineage against a panel of IMC-infected CEM.NKR_CCR5_ cells (AE.CM235, B.WITO, C.TV-1, C.MW965, C.1086C, C.DU151, and C.DU422) (Table 2). The RV144 prime-boost immunization regimen minimally increased ADCC breadth and potency (DH677 UCA ADCC score, −2.32; DH677.1 ADCC score, −2.20 [see Materials and Methods]). Conversely, RV305 boosting substantially increased ADCC breadth and potency (DH677.3 ADCC score, 4.56) (Fig. 2). These data indicate that the RV144 prime-boost regimen was insufficient to fully affinity mature this C1C2-specific B cell clonal lineage. Rather, RV305 trial boosting of this particular RV144 vaccine recipient profoundly enhanced DH677 lineage ADCC breadth and potency.

### Crystal structure of the potent ADCC-mediating MAb DH677.3.

**FIG 3:**
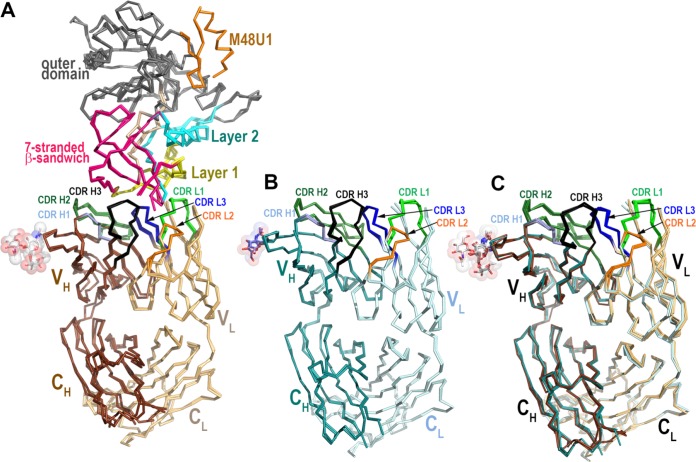
Comparison of the two copies of the DH677.3 Fab-gp120_93TH057_ core_e_-M48U1 complex and the two Fab copies in the apo Fab structure from the asymmetric unit of crystals. (A) The root mean square deviation (RMSD) between complex copies is 0.946 Å for main-chain residues. (B) The RMSD between the Fab copies in the apo Fab structure is 0.540 Å for main-chain residues. (C) Comparison of the free and bound DH677.3 Fab. The α-carbon backbone diagram of superposition of the structures of DH677.3 Fab alone (dark cyan, heavy chain; light cyan, light chain) and N5-i5 Fab bound to CD4-triggered gp120 (dark brown, heavy chain; light brown, light chain). The average RMSD between free and bound Fabs is 0.818 Å for main-chain residues.

**FIG 4:**
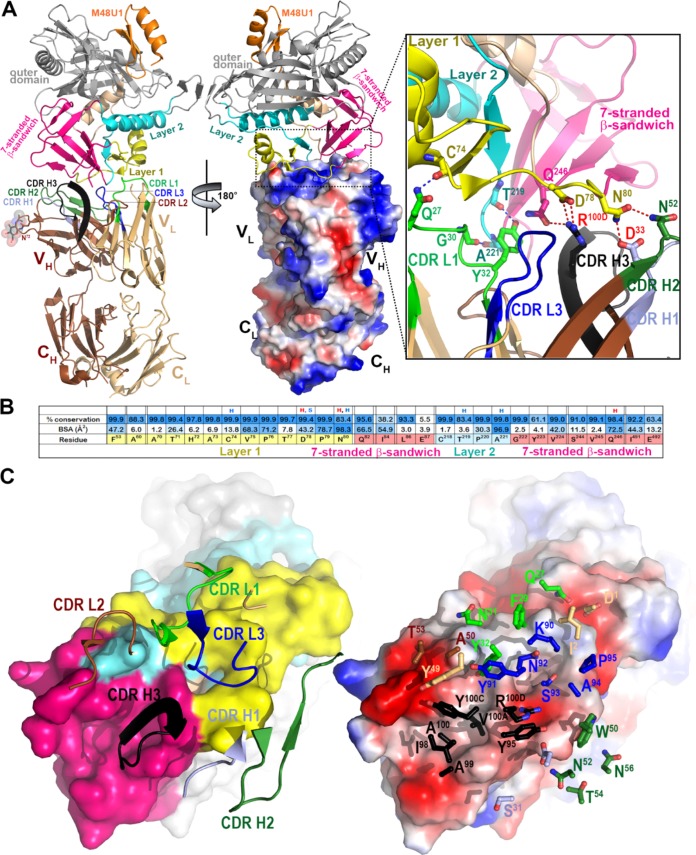
Crystal structure of the DH677.3 Fab-gp120_93TH057_ core_e_-M48U1 complex. (A) The overall structure of the complex is shown as a ribbon diagram (left) with the molecular surface displayed over the Fab molecule (middle), colored based on electrostatic charge: red, negative; blue, positive. The gp120 outer domain is gray and the inner domain colored to indicate inner domain mobile layers 1 (yellow), 2 (cyan), and 3 (light orange) and the 7-stranded β-sandwich (magenta). Complementary determining regions (CDRs) are in the following colors: CDR H1, light blue; CDR H2, dark green; CDR H3, black; CRL1, light green; CDR L2, brown; CDRL3, blue. A blow-up view shows the network of hydrogen (H) bonds formed at the Fab-gp120 interface. H bonds contributed by side-chain and main-chain atoms of gp120 residues are colored magenta and blue, respectively. (B) Fab buried surface area (BSA) and gp120 residues forming DH677.3 epitope are shaded in blue according to BSA (antibody) and percent conservation of gp120 residues (Env). gp120 main-chain (blue) and side-chain (red) hydrogen bonds (H) and salt bridges (S) are shown above the residue. (C) The DH677.3 Fab-gp120_93TH057_ core_e_ interface. CDRs are shown as ribbons (left) and balls and sticks of residues contributing the binding (right) over the gp120 core. The molecular surface of gp120 is colored as described for panel A (left) and by electrostatic potential (right).

**FIG 5:**
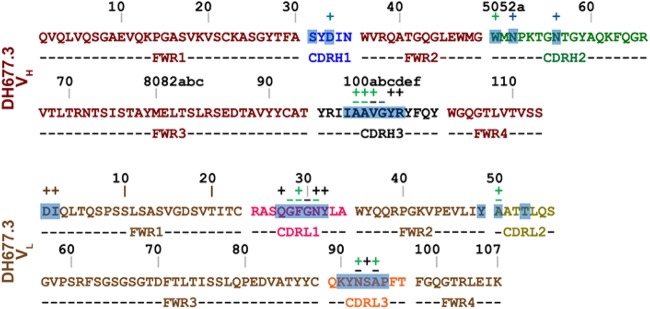
DH677.3 heavy- and light-chain contact residues. MAb side-chain (+) and main-chain (−) contact residues, colored green for hydrophobic, blue for hydrophilic, and black for both, as determined by a 5-Å cutoff value over the corresponding sequence. CDRs are colored as described for Fig. 1, and buried surface residues as determined by PISA are shaded.

We next determined the crystal structure of the antigen binding fragment (Fab) of the highest-ranking RV305 ADCC MAb, DH677.3 (Table 4), alone and in complex with clade AE gp120_93TH057_ core_e_ plus the CD4-mimetic M48-U1 (Fig. 3 and Table 5). DH677.3 Fab-gp120_93TH057_ core_e_-M48U1 complex (Fig. 4) showed that, similar to other cluster A MAbs, DH677.3 approaches gp120 at the face that is buried in the native Env trimer (3–5) and binds the C1C2 region exclusively within the gp120 inner domain. The gp120 residues involved in DH677.3 binding map to the base of the 7-stranded β-sandwich (residues 82, 84, 86 to 87, 222 to 224, 244 to 246, and 491 to 492) and extend into the mobile layers 1 (residues 53, 60, and 70 to 80) and 2 (residues 218 to 221). By docking at the layer 1/2/β-sandwich junction, the Fab buried surface area (BSA) utilizes 248 Å^2^ of the β-sandwich, 542 Å of layer 1, and 135 Å^2^ of layer 2 (Fig. 4 and Table 5). The majority of contacts providing specificity involve a network of hydrogen bonds and a salt bridge (Fig. 4, inset) contributed by the antibody heavy-chain and gp120 side-chain atoms of layer 1 (α turn connecting the β1-β0 strands, D^78^ and N^80^) and the 7-stranded β-sandwich (strand β7, Q^246^). The contacts provided by the light chain are less specific and consist of hydrogen bonds to the gp120 main-chain atoms and hydrophobic contacts within a hydrophobic cleft formed at the layer 1/2/β-sandwich junction (Fig. 4B and C). Overall, DH677.3 utilizes all six of its complementary determining regions (CDRs) and relies approximately equally on both heavy chain and light chain with a total BSA of 973 Å^2^, 498 Å^2^ for the light chain, and 475 Å^2^ for the heavy chain (Fig. 5 and Table 5). Interestingly, 25 of 29 gp120 contact residues are conserved in >80% of sequences in the HIV Sequence Database Compendium (https://www.hiv.lanl.gov/content/sequence/HIV/COMPENDIUM/compendium.html), with 15 of 29 being effectively invariant (>99% conserved) (Fig. 4B).

**TABLE 5:** Details of the DH677.3, A32, and N12-i3 interfaces

Region	BSA (Å^2^) for^*a*^:		
	DH677.3 fab-gp120_93TH057_ core_e_-M48U1	A32 Fab- ID2_93TH057_ (4YC2)	N12-i3 fab-gp120_93TH057_ core_e_+N/C-M48U1 (5W4L)
gp120 total	925	850	803
7/8-stranded β-sheet	248	0	754
Layer 1	542	645	49
Layer 2	135	205	0
Layer 3	0	0	0
Heavy chain total	475	614	711
FWR	0	17	16
CDR H1	16	103	39
CDR H2	84	83	440
CDR H3	375	411	216
Light chain total	498	234	142
FWR	95	0	0
CDR L1	238	126	10
CDR L2	27	0	0
CDR L3	138	108	132
Heavy- and light-chain total	973	848	853

a Details are based on the DH677.3-gp120_93TH057_core_e_ -M48U1, A32 Fab-ID2_93TH057_, and N12-i3 Fab-gp120_93TH057_core_e_+N/C-M48U1 structures, as calculated by the EBI PISA server (http://www.ebi.ac.uk/msd-srv/prot_int/cgi-bin/piserver). The two copies in the asymmetric unit of the DH677.3, A32, and N12-i3 complexes are averaged.

### Comparison of the DH677.3 mode of binding and epitope footprint to cluster A prototype MAbs.

**FIG 6:**
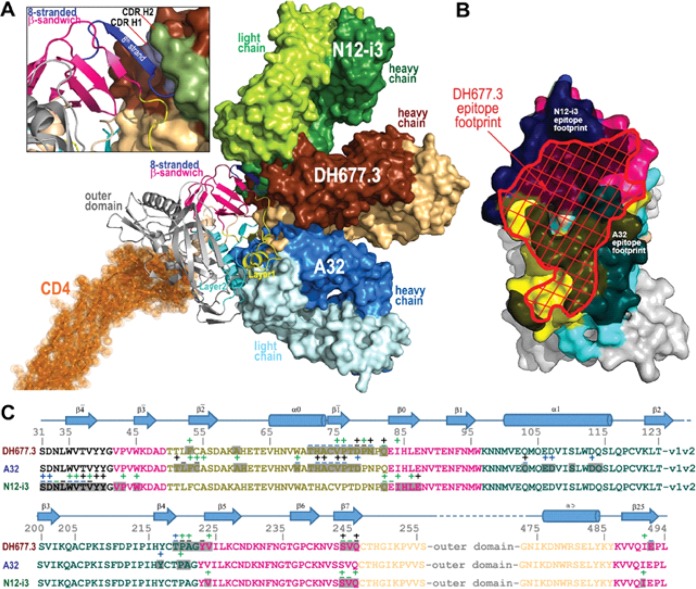
Recognition of HIV-1 Env by DH677.3 and other cluster A MAbs. (A) The overlay of DH677.3 and cluster A MAbs A32 and N12-i3 (C11-like) bound to the gp120 core. Crystal structures of the gp120 antigen in complex with the Fab of DH677.3, A32 (PDB code 4YC2), and N12-i3 (PDB code 5W4L), superimposed based on gp120. The d1 and d2 domains of the target cell receptor CD4 were added to replace peptide mimetic M48U1 of the DH677.3 Fab-gp120_93TH057_ core_e_-M48U1 complex. Molecular surfaces are displayed over Fab molecules and colored in lighter and darker shades of brown, blue, and green for the heavy and light chains of DH677.3, A32, and N12-i3, respectively. A blow-up view shows details of the DH677.3 interaction with the 8-stranded β-sandwich of the gp120 inner domain. The 8th strand (colored in blue) formed by the 11 N-terminal residues of gp120 in the N12-i3 bound conformation (PDB entry 5W4L) was modeled into the DH677.3 Fab-gp120_93TH057_ core_e_-M48U1 complex. CDR H1 and H2 of DH677.3 are colored light blue and dark green, respectively. (B and C) Comparison of DH677.3, A32, and N12-i3 epitope footprints. (B) The DH677.3 epitope footprint (shown in red) is plotted on the gp120 surface with layers colored, as described for Fig. 1, with the A32 and N12-i2 epitope footprints shown in black. (C) DH677.3, A32, and N12-i3 gp120 contact residues are mapped onto the gp120 sequence. Side-chain (+) and main-chain (−) contact residues are colored green for hydrophobic, blue for hydrophilic, and black for both, as determined by a 5-Å cutoff value over the corresponding sequence. Buried surface residues, as determined by PISA, are shaded. The DH677.3 epitope footprint overlays with the epitopes of both A32 and N12-i3.

Antigen complex structures of A32 and N12-i3 (C11-like) (3, 6), MAbs isolated from HIV-1-infected individuals, confirm that DH677.3 recognized a unique epitope between the A32 and C11 antibody-binding sites involving Env epitope elements of both (Fig. 6). While the A32 MAb epitope consists exclusively of gp120 mobile layers 1 and 2 (76% and 24% of gp120 BSA, respectively; Fig. 6 and Table 5), DH677.3 relies less on layers 1 and 2 (53% and 14% of gp120 BSA, respectively) and effectively utilizes the gp120 7-stranded β-sandwich (24% of gp120 BSA) (Fig. 6 and Table 5). The ability to recognize the 7-stranded β-sandwich renders DH677.3 similar to the C11-like antibody N12-i3, which almost exclusively depends on the β-sandwich for binding (94% of its total gp120 BSA; Fig. 6 and Table 5). Interestingly, N12-i3 and other C11-like MAbs require the N terminus of gp120 for binding and recognize a unique gp120 conformation formed by docking of the gp120 N terminus as an 8th strand to the β-sandwich to form an 8-stranded-β-sandwich structure (6). The DH677.3 complex crystals were obtained with gp120_93TH057_ core_e_, which lacks the N terminus (11-aa deletion), and therefore a direct judgment, based on structure, of whether or not the 8th strand is involved in binding was not possible. However, we were able to model the N/C termini-gp120_93TH057_ core_e_ from the N12-i3 Fab complex structure (PDB code 5W4L) to the DH677.3 Fab-gp120_93TH05_7 core_e_-M48U1 complex without any steric clashes (Fig. 6A, inset). Both the conformation and orientation of CDR H1 and H2 of DH677.3 allowed easy access to the 8-stranded β-sandwich structure and enabled contacts to the 8th strand. These data indicated that DH677.3 is capable of accommodating both the 7- and 8-stranded-β-sandwich conformations of gp120 with effective contacts to the 8th strand. Thus, the vaccine-induced C1C2-specific MAb DH677.3 has a unique binding angle to the C1C2 region compared to the infection-induced C1C2-specific MAbs C11 and A32.

### DH677 lineage MAbs mediate ADCC against CD4-downmodulated HIV-1-infected cells.

During natural infection the HIV-1 accessory protein Nef downregulates CD4 expression on the surface of virus-infected cells (14, 15). Cell surface-expressed CD4 facilitates the exposure of CD4i Env epitopes, like C1C2, by binding to coexpressed cell surface Env (16). The analyses of ADCC breadth was performed using target cells infected with IMCs containing the *Renilla* luciferase (LucR) reporter gene, which restricts Nef expression, leading to incomplete CD4 downregulation (17). Nevertheless, Vpu expression can compensate for Nef function and induce CD4 downregulation during the 72-h incubation of the target cells before assays were performed. To exclude any possible impact of this technical aspect of IMCs with LucR on our ADCC results, full-length IMCs (*n* = 7) that do not contain a report gene were used to evaluate ADCC of the affinity-matured RV305 C1C2-specific MAbs DH677.3 and DH677.4 (Fig. 2) and A32 (2). Since clade CRF01_AE possesses a histidine at Env HXB2 position 375 that influences sensitivity to CD4i antibody binding and ADCC (18, 19), only clade B and clade C HIV-1 IMCs were used (Table 2).

**FIG 7:**
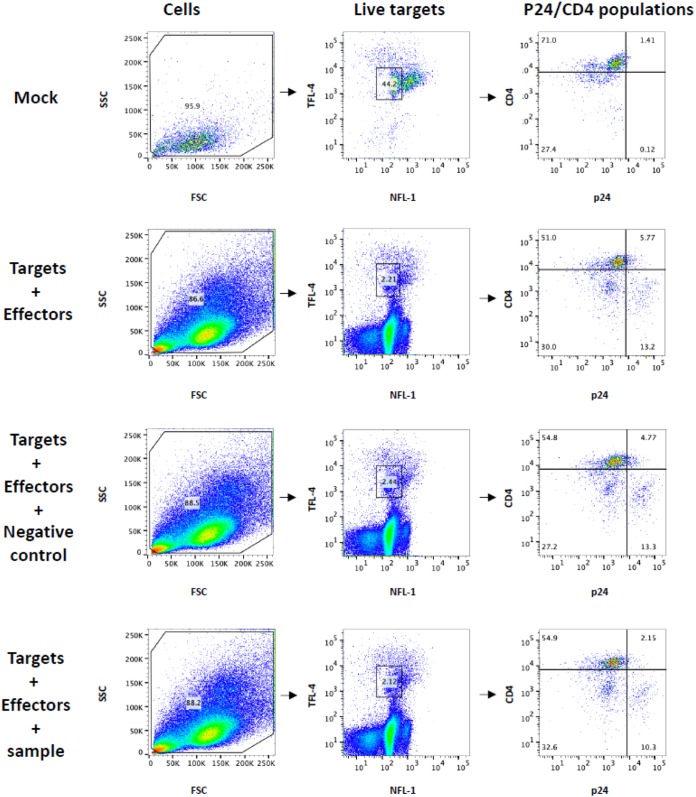
Gating strategy for infected cell elimination assay. Cells are gated on forward scatter and side scatter (FSC and SSC, respectively), followed by distinguishing viable cells using NFL-1 (a dead cell marker) and TFL-4 (target cell marker). Target cells are then gated on CD4 and p24. Mock-infected cells are used to determine positioning of the gate for p24^+^. The proportion of p24^+^, p24^+^ CD4^+^, and p24^+^ CD4^−^ target cells in the presence of effectors only, effectors plus negative control, or effectors plus sample was determined.

**FIG 8:**
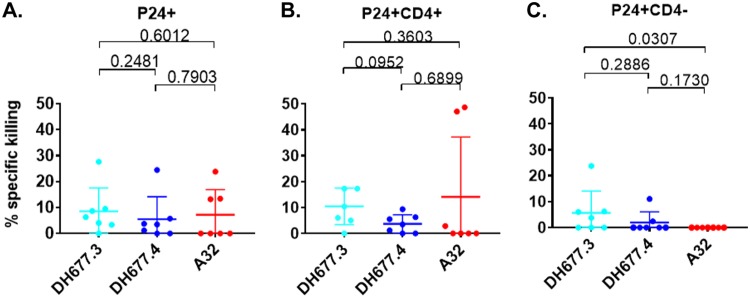
RV305-derived C1C2-specific MAb DH677.3 is significantly better than A32 at mediating antibody-dependent cellular cytotoxicity against CD4 downmodulated infectious molecular clone (IMC)-infected cells. Cells were infected with clade B and clade C full-length IMC that do not contain a reporter gene. Surface CD4 expression was analyzed by flow cytometry, and p24 expression was measured in live/viable, all p24^+^ (A), p24^+^ CD4^+^ (B), and p24^+^ CD4^−^ (C) IMC-infected cell populations. Data are shown with the means and standard deviations.

As these full-length IMCs did not contain a reporter gene, we used an infected cell elimination assay, which measures the reduction of live p24^+^, p24^+^ CD4^+^, and/or p24^+^ CD4^−^ cell populations in the presence of effector cells (Fig. 7). When evaluating elimination of total p24^+^ cells, no significant difference (*P* > 0.05 by Wilcoxon rank sum test) in specific killing was noted among the three MAbs (Fig. 8A). However, when infected cells were separated into p24^+^ CD4^+^ (Fig. 8B) and p24^+^ CD4^−^ (Fig. 8C), it was found that the RV305-boosted DH677.3 MAb mediated ADCC against 4 out of 7 HIV-1 IMCs, whereas DH677.4 and A32 MAb mediated ADCC against two or none of these IMCs, respectively. Although DH677.3 percent specific killing against these IMCs was low (mean, 6%; range, 0 to 24%), the level was significantly higher (*P* = 0.03 by Wilcoxon rank sum test) for mediating ADCC against p24^+^ CD4^−^-infected cells (Fig. 8C) than that of A32. This is likely related to the unique DH677 clonal lineage epitope, which may be more frequently exposed on Env conformations on the surface of IMC-infected cells even in the context of CD4 downmodulation.

## DISCUSSION

In this study, it was found that late boosting of RV144 vaccinees increased C1C2-specific MAb V_H_ + V_L_ chain gene mutation frequency and increased clonal lineage-specific ADCC breadth and potency (Fig. 1 and 2 and Table 4). We analyzed the somatic hypermutation frequency of MAbs obtained after boosting and did not observe a clear correlation between somatic hypermutation frequency and ADCC breadth or potency. Boosting likely affinity matures antibody clonal lineages that are capable of acquiring broad and potent ADCC, as well as antibody clonal lineages incapable of acquiring broad and potent ADCC. For example, DH689 clonal lineage members DH689.1 and DH689.2 were both ∼10% mutated (Table 1), but ADCC breadth and potency was worse than that of most of the RV144 MAbs assayed (Table 4). Conversely, boosting increased somatic hypermutation along with ADCC breadth and potency within the DH677 clonal lineage (Fig. 2). Thus, clonal lineages with fine epitope specificities capable of being matured to increased ADCC breadth and potency greatly benefited from the two AIDSVAX B/E boosts given in the RV305 clinical trial. It should be noted that in VAX003 and VAX004 clinical trials ADCC responses peaked at 3 to 4 immunizations and declined after 5 to 7 immunizations (20). Collectively these data indicate that while the RV144 clinical trial was underboosted, repetitive subsequent boosting beyond RV305 does not necessarily lead to continuously better functional antibody outcomes.

Improving vaccine-induced NNAb effector function will also require more detailed immunological studies on the timing and frequency of boosting. In the VAX003 (ClinicalTrials registration no. NCT00002441) and VAX004 (ClinicalTrials registration no. NCT00002441) trials, frequent protein immunizations skewed Env-specific antibody subclass usage from the highly functional IgG3 to IgG4 (21–23). The RV305 boosts that were studied here occurred several years (6 to 8 years) after the final RV144 boost, unlike previous HIV-1 vaccine trials. Whether the boosting interval can be shortened without skewing antibody subclass usage is not known, but it is possible that boosting with long rest intervals (≥1 to 2 years) will be necessary.

The AIDSVAX B/E protein used for boosting in the RV144 and RV305 HIV-1 vaccine trial contained an N-terminal 11-amino-acid deletion. Previously it was shown that this modification enhanced exposure of the C1C2 region and V2 loop (10). Here, we show that this modification disrupts C11-like MAb binding (Fig. 1) but does create a germ line-targeting immunogen for DH677-like B cell lineages (Fig. 2). Ligand crystal structure analysis found that DH677.3 recognized a unique C1C2 epitope that involves epitope footprints of cluster A MAb A32 and N12-i3 (C11-like), as well as new elements of the inner domain layer 1 and the 7-stranded β-sandwich (Fig. 6). The DH677.3 epitope is positioned midway between the A32 and N12-i3 binding sites, with most residues being highly conserved. Interestingly, DH677.3 binds at the edge of the gp120 inner domain 7-stranded β-sandwich and with layers 1 and 2. This binding mode allows it to bind a gp120 conformation emblematic of the late stages of HIV entry recognized by C11 and C11-like MAbs (6). Most likely this allows DH677.3 to recognize a broader range of Env targets, emerging in both early (when the A32 epitope becomes available) and late (when the C11 epitope becomes available) stages of the viral entry process.

Identification of stage 2A of the HIV-1 Env expressed on the surface of infected cells in the presence of the CD4 molecule or CD4 mimetics reiterate the importance of targeting these epitopes by vaccine-induced responses as detected in our assays (24). In addition, a model of DH677.3 in complex with gp120 antigen bound to CD4 of a target/infected cell confirms that the recognition site and angle of approach position the DH677.3 IgG for easy access for effector cell recognition and Fc-effector complex formation (Fig. 6A). Interestingly, we recently characterized JR4, a MAb isolated from a nonhuman primate that, like DH677.3, recognizes an epitope that includes elements of both the A32 and C11 binding sites (5). JR4 uses its CDR H1 to contact layer 2 residues of the A32 epitope region and its CDR H3 to reach the residues of the 7-stranded β-sandwich. However, in contrast to DH677.3, which largely relies on recognizing the 7-stranded β-sandwich, access of JR4 to this region is limited and involves only a few residue contacts. In this regard, JR4 is more like A32, with main anchoring contacts to layer 2 that limit its reach to the 7-stranded β-sandwich. DH677.3 misses these A32 layer 2 residues and shifts its epitope footprint more toward the 7-stranded β-sandwich, placing it midway between the two epitope regions.

ADCC-mediating antibodies have been shown to reduce mother-to-child HIV-1 transmission (25–27) and slow virus disease progression (27–29), and in RV144 they were correlated with reduced risk of infection in vaccine recipients with lower anti-Env plasma IgA responses (7). Synergy between the RV144 C1C2 and V2 MAbs suggest a role for the C1C2 plasma responses that could not be directly identified by the correlates of the protection study. That DH677.3 was better than A32 at mediating ADCC against HIV-1 clade B and C CD4 downmodulated cells (Fig. 8) makes this MAb an attractive candidate for targeting HIV-1-infected cells *in vivo* in the setting of HIV-1 infection. We have previously shown that the C1C2 MAb A32, when formulated as a bispecific antibody, can potently opsonize and kill HIV-1-infected CD4^+^ T cells (30). Whether the DH677.3-type MAbs are superior to A32 for targeting virus-infected cells remains to be determined.

In summary, our data demonstrate that if the RV144 vaccine trial had been boosted, ADCC-mediating C1C2-specific antibodies would have undergone affinity maturation for both ADCC potency and breadth of recognition of HIV-1-infected CD4^+^ T cells.

## MATERIALS AND METHODS

### Ethics statement.

The RV305 clinical trial (ClinicalTrials registration no. NCT01435135) was a boosting of 162 RV144 clinical trial participants (NCT00223080) six to eight years after the conclusion of RV144 (31). Donors used in this study were from groups boosted twice with either AIDSVAX B/E plus ALVAC-HIV (vCP1521) (group I) or AIDSVAX B/E alone (group II). PBMCs were only collected 2 weeks after the second boost. The RV305 clinical trial (NCT01435135) received approvals from the Walter Reed Army Institute of Research, Thai Ministry of Public Health, Royal Thai Army Medical Department, Faculty of Tropical Medicine, Mahidol University, Chulalongkorn University Faculty of Medicine, and Siriraj Hospital. Written informed consent was obtained from all clinical trial participants. The Duke University Health System Institutional Review Board approved all human specimen handling.

### Antigen-specific single-cell sorting.

All PBMCs used in this study were collected 2 weeks after the second boost in RV305. A total of 1 × 10^7^ PBMC per vaccine recipient were stained with AE.A244gp120Δ11 fluorescently labeled proteins and a human B cell flow cytometry panel. Viable antigen-specific B cells (AqVd^−^ CD14^−^ CD16^−^ CD3^−^ CD19^+^ IgD^−^) were single-cell sorted with a BD FACSAria II SORP (BD Biosciences, Mountain View, CA) into 96-well PCR plates and stored at –80°C for RT-PCR.

### Single-cell reverse transcriptase PCR.

Single B cell cDNA was generated with random hexamers using SSIII. The antibody heavy- and light-chain variable regions were PCR amplified using AmpliTaq360 master mix (Applied Biosystems). PCR products were purified (Qiagen, Valencia, CA) and sequenced by Genewiz. Gene rearrangements, clonal relatedness, unmutated common ancestors, and intermediate ancestor inferences were made using Cloanalyst (13). The DH677 clonal lineage tree was generated using FigTree.

### MAb production.

PCR-amplified heavy- and light-chain gene sequences were transiently expressed as previously described (32). Ig-containing cell culture supernatants were used for ELISA binding assays. For large-scale expression, V_H_ and V_L_ chain genes were synthesized (V_H_ chain in the IgG1 4A backbone) and transformed into DH5α cells (GeneScript, Piscataway, NJ). Plasmids were expressed in Luria broth and purified (Qiagen, Valencia, CA), and Expi293 cells were transfected using ExpiFectamine (Life Technologies, Carlsbad, CA) by following the manufacturer’s protocol. After 5 days of incubation at 37°C 5% CO_2_, the Ig-containing medium was concentrated and purified with protein A beads, and the antibody buffer was exchanged into PBS.

### Antibody binding and blocking assays.

Direct ELISAs were performed as previously described (32). Briefly, 384-well microplates were coated overnight with 30 ng/well of protein. Antibodies were diluted and added for 1 h. Binding was detected with anti-IgG-horseradish peroxidase (HRP) (Rockland) and developed with SureBlue reserve TMB one component (KPL). Plates were read on a plate reader (Molecular Devices) at 450 nm. A32-blocking assays were performed by adding the RV305 antibodies followed by biotinylated A32 and detection with streptavidin HRP.

### Neutralization assays.

TZM-bl neutralization assays were performed as previously described (33). No neutralization was detected for the MAbs assayed in this study.

### IMCs.

The HIV-1 reporter viruses used were replication-competent IMCs designed to encode the *env* genes of CM235 (subtype A/E; GenBank accession no. AF259954.1), WITO (subtype B; GenBank no. JN944948), 1086.c (subtype C; GenBank no. FJ444395), TV-1 (subtype C; GenBank no. HM215437), MW96.5 (subtype C), DU151 (subtype C; GenBank no. DQ411851), and DU422 (subtype C; GenBank no. DQ411854) in *cis* within an Nef-deficient isogenic backbone that expresses the *Renilla* luciferase reporter gene (34). The subtype AE Env-IMC-LucR viruses used were the NL-LucR.T2A-AE.CM235-ecto (IMC_CM235_) (plasmid provided by Jerome Kim, US Military HIV Research Program) and clinical *env* IMCs from the RV144 trial that were built on the 40061-LucR virus backbone. All other IMCs were built using the original NL-LucR.T2A-ENV.ecto backbone as originally described (35). Reporter virus stocks were generated by transfecting 293T cells with proviral IMC plasmid DNA, and virus titer was determined on TZM-bl cells for quality control (35).

### Infection of CEM.NKR_CCR5_ cell line with HIV-1 IMCs.

CEM.NKR_CCR5_ cells were infected with HIV-1 IMCs as previously described (36). Briefly, IMCs were titrated in order to achieve maximum expression within 48 to 72 h postinfection, as determined by detection of luciferase activity and intracellular p24 expression. IMC infections were performed by incubation of the optimal dilution of virus with CEM.NKR_CCR5_ cells for 0.5 h at 37°C and 5% CO_2_ in the presence of DEAE-dextran (7.5 μg/ml). The cells were subsequently resuspended at 0.5 × 10^6^/ml and cultured for 48 to 72 h in complete medium containing 7.5 μg/ml DEAE-dextran. For each ADCC assay, we monitored the frequency of infected target cells by intracellular p24 staining. Assays performed using infected target cells were considered reliable if cell viability was ≥60% and the percentage of viable p24^+^ target cells on assay day was ≥20%.

### Luciferase ADCC assay.

ADCC activity was determined by a luciferase-based assay as previously described (8, 37). Briefly, CEM.NKR_CCR5_ cells (NIH AIDS Reagent Program, Division of AIDS, NIAID, NIH, from Alexandra Trkola) (38) were used as targets after infection with the HIV-1 IMCs. PBMCs obtained from an HIV-seronegative donor with the heterozygous 158F/V and 131H/R genotypes for FcγR3A and FcγR2A (39, 40), respectively, were used as a source of effector cells and were used at an effector-to-target ratio of 30:1. Recombinant MAbs were tested across a range of concentrations using 5-fold serial dilutions starting at 50 μg/ml. The effector cells, target cells, and Ab dilutions were plated in opaque 96-well half area plates and were incubated for 6 h at 37°C in 5% CO_2_. The final read-out was the luminescence intensity (relative light units, or RLU) generated by the presence of residual intact target cells that have not been lysed by the effector population in the presence of ADCC-mediating MAb (ViviRen substrate; Promega, Madison, WI). The percentage of specific killing was calculated using the formula percent specific killing = [(number of RLU of target and effector well − number of RLU of test well)/number of RLU of target and effector well] × 100. In this analysis, the RLU of the target plus effector wells represents spontaneous lysis in the absence of any source of Ab. The ADCC endpoint concentration (EC), defined as the lowest concentration of MAb capable of mediating ADCC in our *in vitro* assay, was calculated by interpolation of the MAb concentration that intersected the positive cutoff of 15% specific killing. The RSV-specific MAb palivizumab was used as a negative control.

### ADCC score.

Antibodies were tested across a range of concentrations using 5-fold serial dilutions starting at 50 μg/ml. Since the dilution curves are not monotonic due to prozone effect of MAbs, nonparametric area under the curve (AUC) was calculated using the trapezoidal rule, with activity of less than 15% set to 0%. In this study, we used principal component analysis (PCA) to compute an ADCC score that explains both potency and breadth of the MAbs. This method of using PCA to calculate breadth score for neutralizing antibodies has been used in a previous study (41). Similarly, in 2011 a study published in *Atherosclerosis* aimed to study the effect of various risk factors on increase in carotid and femoral intima-media thickness used PCA to calculate a cumulative risk score (42).

PCA is the most commonly used method to reduce the dimensionality of the data set (43). It uses eigenvector decomposition of the correlation matrix of the variables, where each variable is represented by an HIV-1 IMC in our study. Most of the shared variance of the correlations of ADCC AUC of HIV-1 IMCs is explained by the first principal component (PC1) (41). Ideally, one would want to explain 70% of the variance, but this should not be at the expense of adding principal components with an eigenvalue of less than 1 (44). Eigenvalue is a measure of variance in the data along that principal component (PC), and a larger value would mean that corresponding PC explains a larger amount of variance in the data. The rationale behind this methodology of calculating ADCC score is that MAbs will not target all seven HIV-1 IMCs equally, hence, calculation of breadth score for MAbs needs to account for this variation.

In this study, a panel of 7 HIV-1 IMCs was tested, which implies that our data set has seven dimensions. ADCC activity was measured as AUC. In our analysis, PC1 and PC2 have eigenvalues above 1 and together account for 80.57% variance (Table 6). Scores obtained from the first principal component can be interpreted as a weighted average of the 7 HIV-1 IMCs that would account for both potency as well as breadth of the MAbs (44). Higher PC1 score would mean that MAb has higher breadth as well as potency for ADCC activity. To calculate the ADCC score, the standardized AUC value for each MAb is first calculated for each HIV-1 IMC and then multiplied by factor loading of the corresponding HIV-1 IMCs. Lastly, these products are added together. Factor loadings are the correlation coefficients between PCs and HIV-1 IMCs (42). The ADCC score obtained from PC1 is a weighted average of the standardized AUC, where factor loadings obtained from PC1 are used as weights to calculate the weighted average. Standardized AUC values imply zero mean and unit standard deviation. The AUC values below the value of mean AUC will result in negative PC1 scores.

**TABLE 6:** Eigenvalues and variance explained by principle components^*a*^

PC	Eigenvalue	Difference	Proportion of variance	Cumulative variance
1	5.42464828	4.40337817	0.6781	0.6781
2	1.02127012	0.28980217	0.1277	0.8057
3	0.73146795	0.32157054	0.0914	0.8972
4	0.40989741	0.19988330	0.0512	0.9484
5	0.21001411		0.0263	0.9747

a Shown are eigenvalues of the correlation matrix. Eigenvalue is a measure of variance in the data along a particular principal component (PC). Proportion of variance describes the percentage of variability explained by every principal component. As seen in the table, the first two PCs have eigenvalues of more than 1, and they account for 80.57% of variance. ADCC scores are derived from PC1 scores, which have an eigenvalue of 5.424 and cumulative variance of 67.81%.

### Infection of primary cells with HIV-1 IMCs.

IMCs encoding the full-length transmitted/founder sequence of seven individuals infected with either subtype B or C viruses from the CHAVI acute infection cohort (CH77, CH264, CH0470, CH042, CH185, CH162, and CH236) were constructed as previously described (45, 46) and used to infect primary CD4^+^ cells. To infect cells, cryopreserved PBMCs were thawed and stimulated in R20 medium (RPMI medium [Invitrogen] with 20% fetal bovine serum [Gemini Bioproducts], 2 mM l-glutamine [Invitrogen], 50 U/ml penicillin [Invitrogen], and 50 μg/ml gentamicin [Invitrogen]) supplemented with interleukin-2 (IL-2) (30 U/ml; Proleukin), anti-CD3 (25 ng/ml clone OKT-3; Invitrogen), and anti-CD28 (25 ng/ml; BD Biosciences) antibodies for 72 h at 37°C in 5% CO_2_. CD8 cells were depleted from the PBMCs using CD8 microbeads (Miltenyi Biotec, Germany) according to the manufacturer’s instructions, and 1.5 × 10^6^ cells were infected using 1 ml virus supernatant by spinoculation (1,125 × *g*) for 2 h at 20°C. After spinoculation, 2 ml of R20 supplemented with IL-2 was added to each infection, and infections were left for 72 h. Infected cells were used if viability was >70% and more than 5% of cells were p24^+^.

### Infected cell elimination assay.

HIV-1-infected or mock-infected CD8-depleted PBMCs were used as targets, and autologous cryopreserved PBMCs rested overnight in R10 supplemented with 10 ng/ml IL-15 (Miltenyi Biotec) were used as a source of effector cells. Infected and uninfected target cells were labeled with a fluorescent target cell marker (TFL4; OncoImmunin) and a viability marker (NFL1; OncoImmunin) for 15 min at 37°C, as specified by the manufacturer. The labeling of the target cells with these two markers allowed us to clearly identify only the live viable cells in our gating strategy and exclude artifacts related to the presence of dead cell staining. Cells were washed in R10 and adjusted to a concentration of 0.2 × 10^6^ cells/ml. PBMCs were then added to target cells at an effector/target ratio of 30:1 (6 × 10^6^ cells/ml). The target-effector cell suspension was plated in V-bottom 96-well plates and cocultured with 10 μg/ml of each MAb. Cocultures were incubated for 6 h at 37°C in 5% CO_2_. After the incubation period, cells were washed and stained with anti-CD4-peridinin chlorophyll protein-Cy5.5 (clone OKT4; eBioscience) at a final dilution of 1:40 in the dark for 20 min at room temperature. Cells were then washed, resuspended in 100 μl/well Cytofix/Cytoperm (BD Biosciences), incubated in the dark for 20 min at 4°C, washed in 1× Cytoperm wash solution (BD Biosciences), coincubated with anti-p24 antibody (clone KC57-RD1; Beckman Coulter) to a final dilution of 1:100, and incubated in the dark for 25 min at 4°C. Cells were washed three times with Cytoperm wash solution and resuspended in 125 μl PBS–1% paraformaldehyde. The samples were acquired within 24 h using a BD Fortessa cytometer. The appropriate compensation beads were used to compensate for the spillover signal for the four fluorophores. Data analysis was performed using FlowJo 9.6.6 software (TreeStar). Mock-infected cells were used to appropriately position live cell p24^+/−^ and CD_4_^+/−^ gates.

Specific killing was determined by the reduction in percentage of viable p24^+^ cells in the presence of MAbs after taking into consideration nonspecific killing and was calculated as [% p24(target + effector cells) − % p24(targets + effectors + MAb or plasma)]/[% p24(target + effector cells)].

CH65 (an anti-influenza monoclonal antibody, kindly provided by M. A. Moody) was used as a negative control. To remove background signal, the highest value of percent specific killing induced by CH65 was subtracted from the calculated reduction in percentage of p24^+^ cells, and then negative values were rounded to 0%. As the data were background subtracted, no positivity criteria were applied to the data.

### SPR.

The binding and kinetic rate measurements of gp120 proteins against RV305 antibodies were obtained by surface plasmon resonance (SPR) using the Biacore 3000 instrument (GE Healthcare). SPR measurements were performed using a CM5 sensor chip with anti-human IgG Fc antibody directly immobilized to a level of 9,000 to 11,000 RU (response units). Antibodies then were captured at 5 μl/min for 60 s to a level of 100 to 300 RU. For binding analyses, the gp120 proteins were diluted to approximately 1,000 nM in PBS and injected over the captured antibodies for 3 min at 30 μl/min. For kinetics measurements, the gp120 proteins were diluted from 5 to 750 nM and injected using a high-performance kinetics injection for 5 min at 50 μl/min. This was followed by a dissociation period of 600 s and surface regeneration with glycine, pH 2.0, for 20 s. Results were analyzed using Biacore BiaEvaluation software (GE Healthcare). Negative-control antibody (Ab82) and blank buffer binding were used for double reference subtraction to account for nonspecific protein binding and signal drift. Subsequent curve-fitting analysis was performed using a 1:1 Langmuir model with a local *R*_max_, and the reported rate constants are representative of two measurements.

### Protein preparation and complex crystallization.

DH677.3 Fab alone was grown and crystallized at a concentration of ∼10 mg/ml. The structure was solved by molecular replacement with PDB entry 3QEG in space group P2_1_ to a resolution of 2.6 Å. Clade A/E 93TH057 gp120 core_e_ (gp120_93TH057_ core_e_, residues 42 to 492 [Hxbc2 numbering]), lacking the V1, V2, and V3 variable loops and containing an H375S mutation to allow binding of the CD4 mimetic M48U1 (47), was used to obtain crystals of DH677.3 Fab-antigen complex. gp120_93TH057_ core_e_ was prepared and purified as described previously (3). Deglycosylated gp120_93TH057_ core_e_ was first mixed with CD4 mimetic peptide M48U1 at a molar ratio of 1:1.5 and purified through gel filtration chromatography using a Superdex 200 16/60 column (GE Healthcare, Piscataway, NJ). After concentration, the gp120_93TH057_ core_e_-M48U1 complex was mixed with a 20% molar excess of DH677.3 Fab and again passed through the gel filtration column equilibrated with 5 mM Tris-HCl buffer, pH 7.2, and 100 mM ammonium acetate. The purified complex was concentrated to ∼10 mg/ml for crystallization experiments. The structure was solved by molecular replacement using the DH677.3 Fab and PDB entry 3TGT as the searching models in space group P1 to a resolution 3.0 Å. The final *R*_factor_/*R*_free_ (%) for the Fab structure is 19.9/26.1, and the final *R*_factor_/*R*_free_ for the complex is 21.4/27.4 (Table 7). The PDB codes for the deposited structures are 6MFJ and 6MFP, respectively. In each case the asymmetric unit of the crystal contained two almost identical copies of Fab or the Fab-gp120_93TH057_ core_e_ complex (Fig. 3).

**TABLE 7:** DH677.3 structural data collection and refinement statistics

Parameter	Value(s) for^*h*^:	
	DH677.3 fab	DH677.3 fab-gp120_93TH057_-M48U1
Data collection		
Wavelength, Å	0.979	0.979
Space group	P2_1_	P1
Cell parameters		
*a*, *b*, *c* (Å)	81.7, 70.5, 84.3	63.5, 80.1, 88.7
α, β, γ (°)	90, 98.3, 90	84.8, 82.3, 82.2
Complexes/AU	2	2
Resolution (Å)	50–2.62 (2.67–2.62)	50–3.0 (3.05–3.0)
No. of reflections		
Total	96,040	57,891
Unique	28,247	30,469
*R*_merge_,^*a*^ %	15.0 (39.1)	9.2 (44.2)
*R*_pim_,^*b*^ %	9.6 (25.2)	9.2 (44.2)
*CC*_1/2_^*c*^	0.99 (0.84)	0.99 (0.75)
*I*/σ	13.0 (2.6)	10.2 (1.2)
Completeness, %	98.0 (98.0	89.1 (90.7)
Redundancy	3.4 (3.2)	1.9 (1.9)
Refinement statistics		
Resolution, Å	50.0–2.62	50.0–3.0
*R*,^*d*^ %	19.9	21.4
*R*_free_,^*e*^ %	26.1	27.4
No. of atoms		
Protein	6,569	12,278
Water	140	
Ligand/Ion	28	426
Overall B value (Å^2^)		
Protein	38	102
Water	32	
Ligand/ion	58	112
RMSD^*f*^		
Bond lengths, Å	0.004	0.004
Bond angles, °	1.3	1.3
Ramachandran^*g*^ (%)		
Favored	96.0	86.7
Allowed	3.8	10.3
Outliers	0.2	3.0
PDB code	6MFJ	6MFP

a *R*_merge_ = ∑|*I* − |/∑*I*, where *I* is the observed intensity and the average intensity obtained from multiple observations of symmetry-related reflections after rejections.

b *R*_pim_, defined in reference 1.

c *CC*_1/2_, defined by Karplus and Diederichs (56).

d *R* = ∑║F_o_|− | F_c_║/∑|F_o_ |, where F_o_ and F_c_ are the observed and calculated structure factors, respectively.

e *R*_free_, as defined by Brünger (57).

f RMSD = Root mean square deviation

g Calculated with MolProbity.

h Values in parentheses are for the highest-resolution shell.

### Crystallization and data collection.

Initial crystal screens were done in vapor-diffusion hanging-drop trials using commercially available sparse matrix crystallization screens from Hampton Research (Index), Emerald BioSystems (Precipitant Wizard Screen), and Molecular Dimensions (Proplex and Macrosol Screens). The screens were monitored periodically for protein crystals. Conditions that produced crystals were then further optimized to produce crystals suitable for data collection. DH677.3 Fab crystals were grown from 20% polyethylene glycol (PEG) 3000, 100 mM HEPES, pH 7.5, and 200 mM sodium chloride. DH677.3 complex crystals were grown from 25% PEG 4000 and 100 mM morpholineethanesulfonic acid, pH 5.5. Crystals were briefly soaked in crystallization solution plus 20% 2-methyl-2,4-pentanediol (MPD) before being flash frozen in liquid nitrogen prior to data collection.

### Data collection and structure solution.

Diffraction data were collected at the Stanford Synchrotron Radiation Light Source (SSRL) at beam line BL12-2, equipped with a Dectris Pilatus area detector. All data were processed and reduced with HKL2000 (48). Structures were solved by molecular replacement with Phaser (49) from the CCP4 suite (50). The DH677.3 Fab structure was solved based on the coordinates of the N12-i2 Fab (PDB entry 3QEG), and the DH677.3 complex was then solved with coordinates from the DH677.3 Fab model, gp120 (PDB entry 3TGT), and M48U1 (PDB entry 4JZW). Refinement was carried out with Refmac (51) and/or Phenix (52). Refinement was coupled with manual refitting and rebuilding with COOT (53). Data collection and refinement statistics are shown in Table 7.

### Structure validation and analysis.

The quality of the final refined models was monitored using the program MolProbity (54). Structural alignments were performed using the program lsqkab from the CCP4 suite (50). The PISA (55) webserver was used to determine contact surfaces and residues. All illustrations were prepared with the PyMol Molecular Graphic suite (http://pymol.org) (DeLano Scientific, San Carlos, CA, USA). Conservation of the DH677.3 epitope was calculated using the HIV Sequence Database Compendium (https://www.hiv.lanl.gov/content/sequence/HIV/COMPENDIUM/compendium.html), comparing gp120 residues to those of clade B Hxbc2. Only unique sequences in the database having an equivalent residue at each position were included in the calculated percentage, representing approximately 32,000 sequences on average.

### Statistical methods.

For luciferase-based ADCC assay, background correction was performed by subtracting the highest value of percent specific killing induced by CH65 and then rounding off the negative values to zero. In Fig. 5, positivity cutoff criteria were not applied but background correction was performed.

In order to assess if two groups have different responses, pairwise comparisons between groups was conducted using Wilcoxon rank sum test. Statistical analysis was performed using SAS software (SAS Institute, Inc., Cary, NC).

### Data availability.

The structures determined here were deposited under PDB codes 6MFJ and 6MFP.
